# Vascular Smooth Muscle Cells Contribute to Atherosclerosis Immunity

**DOI:** 10.3389/fimmu.2019.01101

**Published:** 2019-05-17

**Authors:** Desheng Hu, Changjun Yin, Shanshan Luo, Andreas J. R. Habenicht, Sarajo K. Mohanta

**Affiliations:** ^1^Department of Integrated Traditional Chinese and Western Medicine, Union Hospital, Tongji Medical College, Huazhong University of Science and Technology, Wuhan, China; ^2^Institute of Hematology, Union Hospital, Tongji Medical College, Huazhong University of Science and Technology, Wuhan, China; ^3^Institute for Cardiovascular Prevention, Ludwig-Maximilians-University Munich, Munich, Germany

**Keywords:** atherosclerosis, vascular smooth muscle cells, endothelial cells, intima, adventitia, artery tertiary lymphoid organs

## Abstract

Vascular smooth muscle cells (VSMCs) constitute the major cells in the media layer of arteries, and are critical to maintain the integrity of the arterial wall. They participate in arterial wall remodeling, and play important roles in atherosclerosis throughout all stages of the disease. Studies demonstrate that VSMCs can adopt numerous phenotypes depending on inputs from endothelial cells (ECs) of the intima, resident cells of the adventitia, circulating immune cells, hormones, and plasma lipoproteins. This plasticity allows them to perform multiple tasks in physiology and disease. In this minireview, we focus on a previously underappreciated activity of VSMCs, i.e., their impact on atherosclerosis immunity via formation of artery tertiary lymphoid organs (ATLOs).

## Plasticity of VSMCs in Physiology and Disease

**Figure 1:**
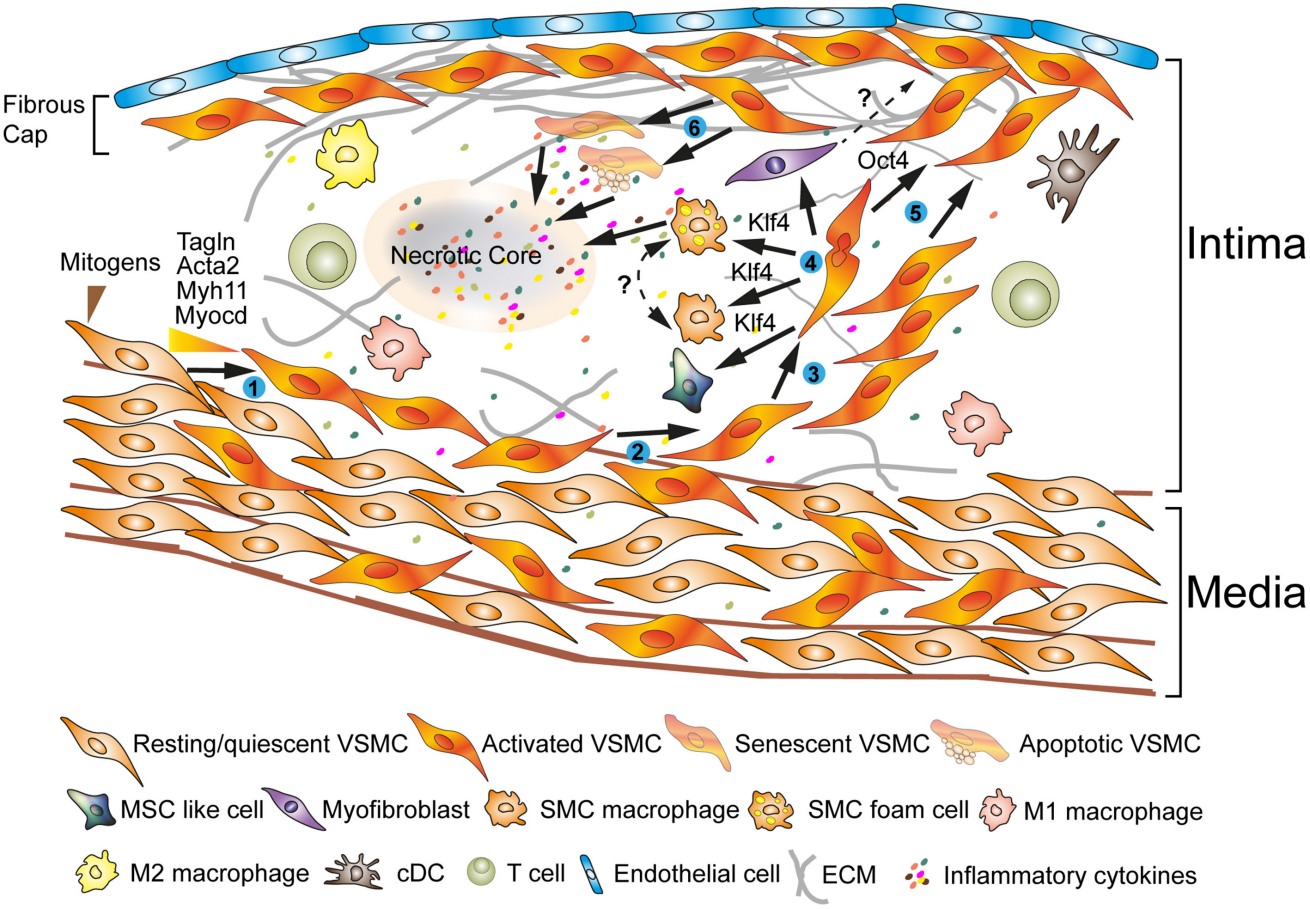
Phenotypes of VSMCs during atherosclerosis progression reveal extraordinary plasticity. ECs and VSMCs in atherosclerotic plaques show activated phenotypes (➊) (3, 4). Some of the VSMCs originate in the media following recruitment into the intima; other VSMCs may be bone marrow-derived or they may originate from myeloid cells in the circulation (➋) (3). A fraction of VSMCs proliferate (➌) (3, 5). VSMCs secrete extracellular matrix components; the pluripotent transcription factor, i.e., Klf4, may play major roles in a process referred to as *phenotype switching* (➍) (6, 7). In attempts to shield the atherosclerotic plaque from lethal rupture, Oct4 may control a process that has been termed remigration to form a fibrous cap (➎) (4, 8–10). Senescence and apoptosis trigger the generation of additional inflammatory cytokines to form a necrotic core initiating a vicious cycle with lethal clinical consequences during the late stages of the disease (➏) (3, 4, 11).

Vascular smooth muscle cells (VSMCs) are the major constituents of medium- and large-sized arteries. Although mechanisms of atherogenesis largely remain to be defined, studies have demonstrated that disease progression involves crosstalk between immune cells with both ECs and VSMCs. Some of these interactions promote plaque growth while others attenuate the size, cellular composition, and stability of atherosclerotic plaques (1, 2) (Figure 1, Table 1). VSMCs show remarkable plasticity in response to vascular injury, inflammation, and lipoprotein accumulation during disease progression via reprogramming gene expression and a shift to a proliferative, pro-migratory, and activated phenotype, i.e., *phenotype switching* (5). In Figure 1, we depict critical molecular switches that have been proposed to be important regulators of disease progression (3, 6, 8, 11). During atherosclerosis initiation, blood-derived monocytes, which have been recruited into the intima, accumulate lipid giving them a foamy appearance. These foam cells contribute to *fatty streak* formation which constitutes the earliest and possibly reversible stage of atherosclerotic plaques. Fatty streaks gradually develop into atheromas/plaques ultimately leading to expanded plaques that contain VSMCs, T cells and myeloid cells (Figure 1) (33). Intriguingly, the composition of an atherosclerotic plaque rather than its size determines its stability as fibrous cap thickness and necrotic core size are potential hallmarks of a stable vs. an unstable plaque, respectively (4, 9). VSMCs in the intima layer are traditionally viewed as beneficial during atherogenesis because they produce extracellular matrix components, thereby promoting formation of stronger fibrous caps resulting in protection against plaque rupture (4, 9). It is increasingly apparent that VSMCs undergo a plethora of structural and functional phenotypical transformations and may even completely lose their native features to acquire characteristics of other cell types including macrophages. Data indicate that VSMCs can acquire dichotomic phenotypes with a Janus head-type nature, i.e., pro- vs. anti-atherogenic properties, depending on the tissue environment and action of risk factors (3). VSMCs release cytokines to stimulate adjacent ECs to express adhesion molecules and release cytokines, and/or enhance chemotaxis of monocyte/macrophages into the plaque (3, 34, 35) (Table 1). These data indicate that accumulation of VSMCs in the fibrous cap or intima are beneficial, whereas their loss or transition into an inflammatory phenotype are detrimental, and that the balance between VSMCs proliferation/migration vs. death/senescence determines atheroprogression vs. plaque stability (3). Various additional aspects of VSMC biology in health and disease were recently covered by a series of excellent reviews (https://academic.oup.com/cardiovascres/issue/114/4) and will not be covered here. Below, we therefore focus attention on the role of the adventitia and the potential impact of VSMCs in ATLO formation.

**Table 1:** Key inflammatory mediators involved in VSMC-mediated atherosclerosis immunity.

	**Cellular sources**	**Cellular targets**	**Roles in SMC dynamics**	**Impact in local immunity**	**Roles in atherosclerosis**	**References**
IL-1α	Apoptotic SMC, EC, T cell, macrophage	SMC, EC, monocyte	Activation, proliferation, remodeling	EC activation, monocyte influx	Damaging	
IL-1β	SMC, macrophage, DC, EC, platelet	SMC, EC, macrophage	Differentiation, proliferation, migration, ECM production, calcification, KLF4 expression	Monocyte/macrophage recruitment and activation, fibrous cap formation, adhesion molecule expression in EC, macrophage polarization	Damaging	(12)
IL-4	Th2 cell, mast cell	SMC, macrophage, Th2 cell	Proliferation	Macrophage proliferation, M2 macrophage polarization, Th2 cell proliferation, and differentiation	Protective	(3)
IL-6	Senescent SMC, EC, T cell, macrophage	SMC, EC, T cell, B cell	Inflammation, apoptosis, calcification	Monocyte influx, myeloid cell differentiation, activation	Controversial	(3, 13)
IL-8	EC, monocyte, T cell	SMC, monocyte, neutrophil, T cell	Inflammation, apoptosis	Leukocyte retention	Damaging	
IL-17	Th17 cell, gd T cell, NK cell, neutrophil	SMC, EC, macrophage, T cell	Inflammation, ECM destabilization	Leukocyte and neutrophil accumulation, EC adhesion molecule expression, MMP release	Controversial	(14)
IL-18	SMC, EC, macrophage	SMC, EC, T cell, macrophage, NK cell	Adhesion molecule expression, ECM remodeling	Adhesion molecule expression, pro-inflammatory cytokine production	Damaging	
IFN-α	SMC, T cell, macrophage	SMC, EC, macrophage	Proliferation, migration, ECM remodeling	Adhesion molecule expression, cytokine production	Damaging	
TNF-α	SMC, T cell macrophage	SMC, monocyte, neutrophil, T cell	ECM production and remodeling, apoptosis, calcification	Neutrophil activation, proinflammatory cytokine production	Damaging	(15)
**Chemokines**						
CCL-2/MCP-1	SMC, EC, macrophage, T cell	SMC, monocyte, neutrophil	ECM remodeling, inflammation, migration	Monocyte recruitment and activation, neutrophil recruitment, pro-inflammatory cytokine release	Damaging	
CCL-19/21	SMC	T cell, macrophage	ECM remodeling	T-cell and B-cell recruitment, proinflammatory cytokine release, macrophage egress	ATLO neogenesis, damaging	(13, 16, 17)
CXCL-12/SDF-1α	SMC, EC, macrophage, platelet	SMC, EC, monocyte, neutrophil	SMC progenitor recruitment, ECM remodeling	EC proliferation, adhesion molecule expression in EC, circulating progenitor recruitment, monocyte influx, plasma cell survival, neutrophil homeostasis, plaque stability	Protective	(18, 19)
CXCL-13	SMC, macrophage	SMC, B cell, macrophage	Anti-apoptosis	B cell recruitment, maturation, proliferate, survival and affinity maturation, macrophage apoptosis, and polarization	ATLO neogenesis, Protective	(16, 20, 21)
CX3CL-1	SMC, EC	SMC, T cell, monocyte, platelet	migration, survival	Monocyte/macrophage and T cell recruitment and adhesion, monocyte survival, platelet–monocyte complex formation	Damaging	
MIF	SMC, EC, macrophage, T cell	SMC, monocyte	Migration, EMC production, and remodeling	Monocyte influx	Damaging	
**Growth factors**						
IGF-1	SMC, EC	SMC, EC, macrophage	Proliferation, migration, apoptosis, ECM production	EC migration, survival, adhesion molecule expression, macrophage chemotaxis	Protective	(22)
PDGF-BB/DD	SMC, EC, platelet macrophage	SMC, EC, fibroblast	Differentiation, proliferation, migration, maturation, ECM production, KLF4 expression, autophagy, survival	EC dysfunction, leukocyte accumulation	Damaging	(23, 24)
TGF–β	SMC, EC, T cell, B cell, macrophage	SMC, EC, Treg, B cell	Differentiation, proliferation, migration, ECM production	Fibrous cap remodeling, EC activation, angiogenesis, anti-inflammatory cytokine production, wound healing	Protective	(25, 26)
**Others**						
Lymphotoxin (TLO inducing cytokine)	Ly6C^hi^ monocyte, T cell, LTi cell	SMC, EC, macrophage, T cell, B cell	SMC activation, trans-differentiation	Angiogenesis, stromal cell differentiation, leukocyte accumulation	TLO neogenesis, protective	(16, 27, 28)
CD-40L (costimulatory molecule)	SMC, EC, T cell, platelet	SMC, EC, macrophage, T cell	Activation, inflammation	Proinflammatory cytokine production, macrophage polarization, MMP release	Damaging	(13)
NLRP-3 (inflammasome)	SMC, macrophage	SMC, macrophage, neutrophil	ECM remodeling, inflammation	Macrophage priming, pro-inflammatory cytokine release	Damaging	(29)
Leukotrien-B4 (lipid mediator)	Macrophage, foam cell, dendritic cell	SMC, EC, macrophage	Proliferation, migration, ECM remodeling	EC activation, leucocyte chemotaxis, and activation	Damaging	
Annexin-A1 (lipid mediator)	EC, macrophage, neutrophil	SMC, neutrophil, T cell, macrophage	Migration, ECM production	Neutrophil and monocyte recruitment, apoptosis, phagocytosis, T cell activation, M2 macrophage, polarization	Protective	(13, 30)
Ephrin-A2 (guidance molecule)	SMC, EC, macrophage	SMC, EC, macrophage	proliferation, ECM production	Angiogenesis, fibrous cap thickness, monocyte adhesion	Damaging	
Netrin-1 (guidance molecule)	SMC, EC, macrophage	SMC, EC, macrophage	Migration, ECM remodeling	EC NO release, macrophage retention, leukocyte trafficking	Protective	(31, 32)

## VSMCs Affect Adventitia Immune Responses in Hyperlipidemic Mice

**Figure 2:**
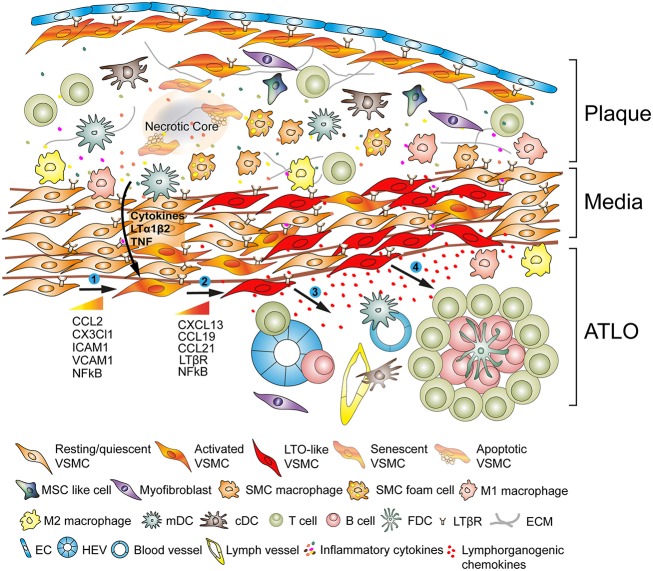
Vascular smooth muscle cells (VSMC) participate in adventitia immunity during plaque formation. VSMCs sandwiched between atherosclerotic plaques and the adventitia adopt a lymphoid tissue organizer-like phenotype following activation via plaque-derived cues (➊) and subsequently transdifferentiate into LTo-like cells (➋). By means of their proliferative and cytokine-expressing phenotype they affect the restructuring and sculpting of the adventitia including angiogenesis, HEV formation, and lymph vessel neogenesis (➌). *Phenotype switching* also results in the expression and secretion of lymphorganogenic chemokines, i.e., CXCL13 and CCL21, thereby promoting ATLO formation depicted schematically in the lower part of the graph (➍).

During the last two decades, it became increasingly apparent that the adventitia is a highly complex and immunologically active tissue harboring cells as diverse as stromal cells, nerves, lymph vessels, vasa vasora, and resident leukocytes/progenitor cells all of which have the ability to affect disease progress (36, 37). We observed that the adventitia of *ApoE*^−/−^ mice undergoes major restructuring events during all stages of atherosclerosis: Both innate and adaptive immune cells accumulate adjacent to the neighboring atherosclerotic plaques (Figure 2). Moreover, our studies in experimental mice were corroborated in human diseased arteries: We and others observed ATLO-like structures in the adventitia of patients presenting with atherosclerotic aortic aneurysms (38, 39) and more recently, Akhavanpoor et al. observed well-developed ATLOs in the adventitia of a considerable percentage of patients afflicted with ischemic heart disease (40). In murine atherosclerosis, lymph vessel-, high endothelial venule (HEV)-, and blood vessel neogenesis are prominent features of this restructuring process (16, 41).

## Aging or Chronicity or Both?

Importantly, aging was found to be a major determinant of adventitia restructuring and sculpting. Fully developed ATLOs form during advanced stages of atherosclerosis but their early stages, i.e., T/B cell aggregates, emerge in parallel to the formation of atherosclerotic plaques (16, 27, 41, 42). Indeed, these early ATLOs form at the age of ~30 weeks and reach more advanced stages at around 52 weeks to peak at 78 weeks (16, 41). Similar age-dependent developmental stages of TLOs have been observed in other chronic unresolvable diseases (1, 42). These data indicate that ATLO development is age-dependent and aging is positively associated with adventitial sculpting (Figure 2). However, the relation between aging and chronicity is difficult to study in murine atherosclerosis as atherosclerotic plaque development begins in the aortic arch, then travels sown the aorta to reach the abdominal aorta at later disease stages thus taking extended periods of time. More work is needed to clarify the important issue whether immune cell and/or VSMC senescence contributes to ATLO formation and which cells types, i.e., VSMCs, ECs, and/or immune cells are involved. Studies in other forms of TLO formation such as those occurring in the lung and the gastrointestinal tract would indicate that it is the chronicity of a persistent inflammatory tissue reaction rather than *bona fide* aging events of the immune system that is crucially contributing to the formation of ATLOs (43, 44).

## Newly Formed Adventitia Structures Participate in atlo Function by Promoting Lymphocyte Recirculation

Advanced ATLO stages include T/B cell aggregates, germinal centers within activated B cell follicles, lymph vessels, HEVs in T cell areas, and extracellular conduit meshworks (16). These major changes resemble structures reminiscent of those found in secondary lymphoid organs where they promote fundamental aspects of immune responses toward antigen including recirculation of naïve lymphocytes to find their cognitive antigens/autoantigens, and organizing affinity maturation of B cells toward potential autoantigens. Using adoptive lymphocyte transfer studies, we observed that ATLOs greatly enhance lymphocyte recruitment into the arterial wall by both promoting immigration and concomitant attenuation of emigration of antigen-inexperienced lymphocytes (27). Subsequent to their recruitment, T cells become activated, begin to proliferate, and some of the CD4 T helper cells are converted into induced T regulatory cells. These data reveal that the immune system in the adventitia is highly responsive toward the underlying inflammatory tissue reaction of the arterial wall. Moreover, B cells which form germinal centers in activated B cell follicles appear to undergo a germinal center reaction in the presence of follicular dendritic cells where some are converted into memory B cells while others leave the germinal centers to become plasma cells (45). When taken together, these data indicate that ATLOs rather than secondary lymphoid organs (as previously thought) organize atherosclerosis immunity.

## Atlo Formation is Highly Territorialized Indicating that Signals are Transmitted From Plaques to the Adventitia via Lymphoid Tissue Organizer-Like VSMCs

Another aspect of adventitia restructuring during atherogenesis is that ATLO formation is highly territorialized being largely restricted to adventitia segments adjacent to atherosclerotic plaques in the abdominal aorta in mice (16, 45). ATLOs are only occasionally found in the adventitia of the innominate artery or of the aortic arch where atherosclerosis is most prominent. Immune cells in the thoracic segments of the aorta are mainly composed of T cell aggregates compared to ATLOs in the abdominal aorta (16, 41). The exact mechanism for territoriality of ATLOs formation in the abdominal aorta is still unknown. TLOs appear to be a feature of many chronic unresolvable inflammatory diseases and are prominent hallmarks of autoimmune diseases (42). The cellularity and structures of TLOs in atherosclerosis are similar to TLOs in many other chronic diseases including cancer-associated TLO formation (46, 47). The field of TLO biology has dramatically expanded in recent years raising the important possibility that new therapeutic target may be identified via understanding of TLO's function in each of TLO-associated diseases. However, the various types of TLOs reveal some disease-specific features, which may ultimately determine whether the associated immune responses are harmful or protective. Such disease-specific characteristics possibly arise through one of several mechanisms including organ specificity and the nature of tissue damage (1, 42). The development of lymphoid organs is a complex process which involves hematopoietic *lymphoid tissue inducer* (LTi) cells, non-hematopoietic stromal *lymphoid tissue organizer* (LTo) cells and LTβR signaling. Our *in-vitro* studies of mouse aorta VSMCs indicated that upon appropriate stimulation they can serve as LTo-like cells (20), originally identified during embryonic development during the formation of secondary lymphoid tissues including lymph nodes and spleen (48, 49) (see below). It is important to note that ATLOs appear to involve VSMCs as important participants whereas other TLOs involve other LTo-like mesenchymal cells (50). The common denominator of all forms of TLOs, however, appears to be–unlike secondary lymphoid organs–a chronic inflammatory tissue reaction which drives the immune system to form these lymphoid structures close to or in some instances within the diseased tissue (42, 43).

## VSMCs Directly Contribute to Adventitia Immunity in Atherosclerosis

In view of the highly territorialized nature of ATLOs adjacent to atherosclerotic plaques, we reasoned that VSMCs may be involved in ATLO formation and by the same token may adopt a functional role in atherosclerosis progression. VSMCs highly express the LTβR constitutively (16), whereas its ligand LTα_1_β_2_ is expressed on various immune cells termed LTi cells (51). VSMCs are hypothesized to be activated through the LTβR-LT signaling pathway by LTi cells involved in secondary lymphoid tissue neogenesis. The origin of LTi cell has not yet been clearly determined though activated macrophages and other immune cells in the intima plaque are candidates for this activity in LTO neogenesis. In response to cellular and soluble mediators VSMCs appear to undergo a distinct type of phenotype switching to a LTo-like phenotype by paracrine secretion of the lymphorganogenic chemokines, i.e., CXCL13 and CCL21, thereby attracting immune cells, e.g., macrophages/dendritic cells, T cells, and B cells to the local adventitia milieu leading to formation of ATLO in the adventitia (27, 41, 45) (Table 1). Global or VSMC-specific deficiency of LTβR in aged hyperlipidemic *ApoE*^−/−^ mice demonstrated increased atherosclerotic plaque formation indicating that the VSMC LTβR has the ability to attenuate development of atherosclerosis under some experimental conditions (27). Other studies, however, showed that young global *ApoE*^−/−^ *LTbR*^−/−^mice maintained under a high fat diet (HFD) revealed a lower aortic plaque burden than their normal diet-fed *ApoE*^−/−^ counterparts (52). These apparent contradictory data indicate that the roles of LTβR in young mice and under conditions of excessive hyperlipidemia may be different in the two models. Alternatively, the high fat diet which leads to dramatic and possibly intoxicating levels of plasma lipids, may be responsible for these differences. Since we do not regularly use a high fat diet in ApoE^−/−^ mice, these discrepancies remain unresolved issues though it has been shown that the immune system may be overwhelmed by extreme levels of plasma lipids [reviewed in Mohanta et al.(42)]. These data call for further studies to examine the molecular basis for the apparent dichotomy of the LTβR in atherosclerosis progression in young vs. aged mice and/or early vs. advanced atherosclerosis. In addition, bone marrow-derived macrophages may function as LTβR-independent LTi cells and trigger the expression of CCL19, CCL20, and CXCL16 by VSMCs (53) (Table 1). Thus, VSMCs may participate in the formation of TLOs in atherosclerosis by upregulation of lymphorganogenic chemokines to promote immune cell aggregates in the adventitia. As mentioned above, ATLOs are common in humans burdened by ischemic coronary heart disease (40) and in human atherosclerotic aortic aneurysms (38, 39). When taken together, the occurrence of ATLOs in experimental mice and in human atherosclerosis raise important questions regarding the nature of atherosclerosis as an autoimmune disease: it is conceivable that during atherogenesis autoimmune T cells and autoimmune B cells directed against yet to be identified autoantigens are generated. Isolation of such autoimmune lymphocytes including sequencing of their T cell receptors and/or B cell receptors would allow to test major hypotheses in atherosclerosis. Indeed, atherosclerosis research would undergo a major shift and testing of individual autoimmune lymphocyte clones in functional *in vitro* and *in vivo* systems would become possible.

## Conclusion

Atherosclerosis is an inflammatory disease of arteries. VSMCs communicate with both the ECs and immune cells in the atherosclerotic plaques and resident cells in the adventitia. This ability allows VSMCs to affect atherosclerosis immunity in major ways including the formation of ATLOs. Further work is required to delineate the role of VSMCs during different stages of atherosclerosis, determine their role in young vs. aging arteries, and elucidate further their dichotomic roles in disease progression. A better understanding of these processes may open the way to develop therapeutic strategies for future intervention in clinically important late-stages of the disease.

## Author Contributions

All authors contributed to the design, writing, and editing of the submitted manuscript, and approved it for publication.

### Conflict of Interest Statement

The authors declare that the research was conducted in the absence of any commercial or financial relationships that could be construed as a potential conflict of interest.
